# Geometric Stability and Lens Decentering in Compact Digital Cameras

**DOI:** 10.3390/s100301553

**Published:** 2010-03-01

**Authors:** Enoc Sanz-Ablanedo, José Ramón Rodríguez-Pérez, Julia Armesto, María Flor Álvarez Taboada

**Affiliations:** 1 Geomatics Engineering Research Group, University of León, Avda. Astorga s/n, 24400 Ponferrada, Spain; E-Mails: jr.rodriguez@unileon.es (J.R.R.-P.); flor.alvarez@unileon.es (M.F.A.T.); 2 Department of Natural Resources and Environmental Engineering, University of Vigo, Campus Universitario As Lagoas-Marcosende s/n, 36200 Vigo, Spain; E-Mail: julia@uvigo.es

**Keywords:** geometric stability, digital camera, photogrammetry, lens decentering

## Abstract

A study on the geometric stability and decentering present in sensor-lens systems of six identical compact digital cameras has been conducted. With regard to geometrical stability, the variation of internal geometry parameters (principal distance, principal point position and distortion parameters) was considered. With regard to lens decentering, the amount of radial and tangential displacement resulting from decentering distortion was related with the precision of the camera and with the offset of the principal point from the geometric center of the sensor. The study was conducted with data obtained after 372 calibration processes (62 per camera). The tests were performed for each camera in three situations: during continuous use of the cameras, after camera power off/on and after the full extension and retraction of the zoom-lens. Additionally, 360 new calibrations were performed in order to study the variation of the internal geometry when the camera is rotated. The aim of this study was to relate the level of stability and decentering in a camera with the precision and quality that can be obtained. An additional goal was to provide practical recommendations about photogrammetric use of such cameras.

## 1. Introduction

In recent years, improved resolution and sensitivity of photo sensors and decreasing costs have enabled the emergence of novel photogrammetric applications performed with many types of non-metric cameras. Low-cost cameras, amateur cameras, semi-professional and professional cameras are being used for field applications [1], structural surveying [2], structural engineering [3], materials sciences [4], measurement systems [5,6] and aerial mapping [7].

These cameras, not initially designed for metric purposes [8], have technologies such as autofocus, zoom lenses, retrofocus construction and image stabilizers, which can potentially reduce their accuracy [9]. In addition to these technologies, many of which can be disabled, the major limitation of non-metric cameras is their low geometric stability, e.g., the low reliability and durability of the camera’s internal geometry over time and even between successive images [10].

Some studies have been conducted to determine the variations occurring in camera internal geometry as a result of instability, along with the origin of the instability itself. For example, Shortis *et al.* [10] report on an investigation into the physical behavior of the principal point location and then compares different calibration parameter models for the Kodak DCS420 and DCS460 digital still cameras. Results showed that the response of the CCD array to camera roll is the most evident source of systematic error. Shortis *et al.* [11] report the effect of handling (including shaking repeatedly to simulate rough handling and repeated powered power cycling to test the repeatability of the zoom and focus settings), and remedial measures to determine the stability of the principal point’s location were conducted. In [12] examinations centered upon the stability of the camera back with respect to the camera body were done. In this work, Mills *et al.* analyzed the position of the principal point, the principal distance and distortion parameters. Calibration tests on a range of different digital cameras, all within the SLR class, were conducted by Shortis *et al.* [13] to ascertain the differences between zoom and fixed lenses used with these cameras. The analyses presented indicate that there are differences between the two lens types in terms of accuracy, precision and stability, suggesting that although acceptable results can be obtained using zoom lenses, a fixed lens provides superior results. Wackrow *et al.* [14] studied the geometric stability and manufacturing consistency was obtained from seven identical low-cost digital cameras (Nikon Coolpix 5400) over a one year period. The study examined the degree of similarity between interior parameters of the cameras. The variation in radial lens distortion was also analyzed. With these cameras, an accuracy of 1.4 mm from a distance of 1.5 m was achieved.

The research presented here studied the variation of the internal geometry parameters (principal distance, principal point position and parameters that define lens distortion) in various situations and in six theoretically identical digital cameras. We also quantified the decentering present in each sensor/lens set, as related to the respective geometric stability and the photogrammetric precision that can be obtained from each one.

Four of these cameras are part of medium precision 3D measuring equipment used to measure living beings [6]. This camera model meets the required characteristics for this application: low weight, high resolution, and above all the possibility of remote shooting. By contrast, the retractile nature of its lenses and compact design prevents the mechanical stabilization [15] of the sensor-lens system. The intent of this paper is to identify the internal geometry changes that occur in the cameras during normal use. This knowledge should be able to provide criteria on the optimal use of them, including frequency calibration, the need to keep the camera alive between different photos, convenience of using the camera in a single position, and so on.

## 2. Modeling and Calibration of Cameras

The purpose of modeling the cameras in the context of photogrammetric metrology is to obtain a theoretical model that describes how a scene is transformed into an image [16]. As a result of modeling, the real camera is idealized or simplified to express its behavior using mathematical expressions, which ultimately enable its metric uses. The performance of the measurement system depends largely on the accuracy of the modeling.

**Figure 1.**
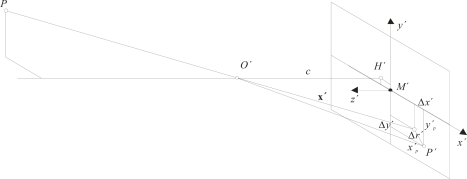
Interior orientation parameters [17].

Figure 1 illustrates all internal parameters used in modeling of this work. Position and distance of the perspective center and deviations from the central perspective model are described with respect to the image coordinate system, as defined by the pixel array. The origin of the image coordinate system is located in the image plane and coincides with the perspective center. Hence, *H*′ is the principal point, the nadir of the perspective center *O*′ with image coordinates (*x*′_0_, *y*′_0_) approximately equal to the center of the image *M*′. The principal distance, *c*, is the normal distance to the perspective center from the image plane and is approximately equal to the focal length *f* when focused at infinity. Parameters of functions describing imaging errors are dominated by the effect of the radial-symmetric distortion Δ*r*′ [17].

When these parameters are known, the (error-free) imaging vector **x**′ can be defined with respect to the perspective center (hence, the principal point):

(1) $$x^{\prime }=\left[\begin{array}{c} x\prime \\ y\prime \\ z^{\prime } \end{array}\right]=\left[\begin{array}{ccc} x_{p}^{\prime } & -x_{0}^{\prime } & -\Delta x\prime \\ y_{p}^{\prime } & -y_{0}^{\prime } & -\Delta y\prime \\ & -c & \end{array}\right]$$

where *x*′*_p_*, *y*′*_p_* are the measured coordinates of image point *P*′; *x*′_0_, *y*′_0_ are the coordinates of the principal point *H*′; and Δ*x*′, Δ*y*′ are the axis-related correction values for image errors.

Deviations from the ideal central perspective model, attributable to image errors, are expressed in the form correction functions Δ*x*′, Δ*y*′ with respect to the measured image coordinates. In the first instance, measured image coordinates *x*′*_p_*, *y*′*_p_* are corrected by an offset of the principal point *x*′_0_, *y*′_0_:

(2) $$\begin{array}{l} x{}^{\circ}=x_{p}^{\prime }-x_{0}^{\prime }\\ y{}^{\circ}=y_{p}^{\prime }-y_{0}^{\prime } \end{array}$$

Hence, the image coordinates *x*°, *y*° are corrected by *x*′ = *x*° − Δ*x*′ and *y*′ = *y*° − Δ*y*′. Strictly speaking, the values *x*°, *y*° are only approximations because the corrections Δ*x*′, Δ*y*′ must be calculated using the final image coordinates *x*′, *y*′. Consequently, correction values must be applied iteratively.

Radial (symmetric) distortion constitutes the major imaging error for most camera systems and is attributable to variations in refraction in the lens system. The radial distortion is usually modeled with a polynomial series using the radial distortion parameters *K*_1_ and *K_n_* [18]:

(3) $$\Delta r_{\mathrm{rad}}^{\prime }=K_{1}{r\prime }^{3}+K_{2}{r\prime }^{5}+K_{3}{r\prime }^{7}+...$$

where $r\prime =\sqrt{{x{}^{\circ}}^{2}+{y{}^{\circ}}^{2}}$ is the image radius (*i.e.,* the distance from the principal point). The software used in this work (Photomodeler 6.0) utilizes the following unbalanced variation [19]:

(4) $$\Delta r_{\mathrm{rad}}^{\prime }=r(k_{1}{r\prime }^{2}+k_{2}{r\prime }^{4}+k_{3}{r\prime }^{6}...)$$

Then, the image coordinates are corrected proportionally:

(5) $$\Delta x_{\mathrm{rad}}^{\prime }=x\prime \frac{\Delta r_{\mathrm{rad}}^{\prime }}{r\prime }\;\;\;\;\;\;\;\;\;\;\;\;\Delta y_{\mathrm{rad}}^{\prime }=y\prime \frac{\Delta r_{\mathrm{rad}}^{\prime }}{r\prime }$$

Radial-asymmetric distortion, often called tangential or decentering distortion, is mainly caused by decentering and misalignment of the lens and can be compensated by the following function [18]:

(6) $$\begin{array}{l} \Delta x_{\tan}^{\prime }=B_{1}({r\prime }^{2}+{2x\prime }^{2})+2B_{2}x\prime y\prime \\ \Delta y_{\tan}^{\prime }=B_{2}({r\prime }^{2}+{2y\prime }^{2})+2B_{1}x\prime y\prime \end{array}$$

The radial component of the decentering distortion can be calculated by:

(7) $$\Delta x_{\tan\_\mathrm{rad}}^{\prime }=\Delta x_{\tan}^{\prime }\;\text{cos}\left(\text{arctan}(y/x)\right)+\Delta y_{\tan}^{\prime }\;\text{sin}\left(\text{arctan}(y/x)\right)$$

while the tangential component is:

(8) $$\Delta x_{\tan\_\tan}^{\prime }=\Delta x_{\tan}^{\prime }\;\text{sin}\left(\text{arctan}(y/x)\right)+\Delta y_{\tan}^{\prime }\;\text{cos}\left(\text{arctan}(y/x)\right)$$

The individual terms used for modeling the imaging errors of typical photogrammetric imaging systems can be summarized as follows:

(9) $$\begin{array}{l} \Delta x\prime =\Delta x_{\mathrm{rad}}^{\prime }+\Delta x_{\tan}^{\prime }\\ \Delta y\prime =\Delta y_{\mathrm{rad}}^{\prime }+\Delta y_{\tan}^{\prime } \end{array}$$

The procedure by which a camera is modeled is called calibration. During calibration, a system of equations is obtained that can include the parameters for the interior orientation of a camera as unknowns, including the parameters of functions that describe imaging errors. The system of equations is then solved by minimizing errors via a procedure called bundle adjustment.

## 3. Experimental Design

### 3.1. Cameras Calibration

Six identical Pentax Optio A40 cameras were analyzed (Photograph 1). This camera, announced in October 2007 with a recommended price under US $300, is a typical compact digital camera with a 3× zoom feature and a 1/1.7″ sensor (7.6×5.7 mm) with 12×10^6^ effective pixels. Among the most outstanding characteristics of this compact camera model (from a photogrammetric point of view), is the manual control of aperture and exposure time and the memory storage for position of the zoom and focus when the camera is turned off. This camera also allows manual focus control, which involves the ability to set the lens focus. This is useful with objects at medium or large distances (greater than 4–5 m) since it is easier when the object is entirely within the depth of field. With small objects at short distances (0.5–3 m) it is sometimes preferable to ensure the correct focusing at the expense of allowing small changes in the position of the focusing lens, which inevitably involves small variations in the internal geometry of the camera. The technical specifications of these cameras are given in Table 1.

All photographs made in this work were performed with sensitivity ISO100 and an aperture of F/2.8 with enough ambient light to take photographs using exposure times equal to or less than 1/80s. To ensure a homogeneous level of sharpness in all pictures and for all cameras, autofocus was used in every image and in a restricted area at the center of the scene. The measurement of light to calculate the exposure time was also set using a restricted area at the center of the scene. The maximum resolution and minimum compression JPG format was used, and the optical and digital stabilization features were turned off.

The cameras were repetitively modeled by field calibration using a plane point field (Photograph 2). The field calibration consisted of 144 points of 5 mm diameter and a separation between rows and columns of 80 mm. Four points had two concentric rings whose discontinuities represent a coding system that allows automatic referencing of homologous points. Subpixel detection algorithms were used for detection of the targets in the images.

**Figure 2.**
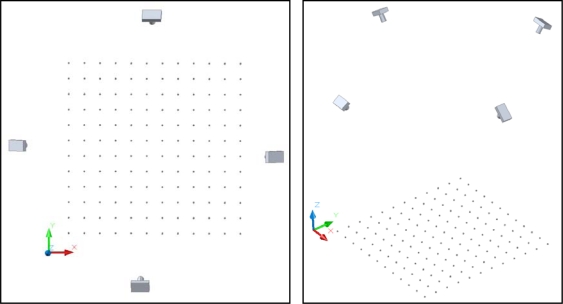
Network configuration of the photogrammetric survey.

For each calibration, 12 convergent images were taken from four camera stations (Figure 2). At each station, the camera was rotated around its optical axis by 0°, 90° and −90°. To study the variation of the internal geometry of the camera position with respect to gravity three subnets were used: four images taken at 0°, four images taken at 90° and four images at −90°. To calculate the position of principal point precisely to the subnet of photographs taken with the camera at 0°, another two photos were added at −90° and 90°. To the other subnets two photos at 0° were added.

The geometric mean distance between the centers of projection and the center of the field calibration was 1.4 m. The incidence angle between opposite pairs of optical axes was of 52°. For the orientation of the model, the points i_2,2_ and i_2,10_ (X axis) and i_2,2_, i_10,2_ (Y axis) were used. For scaling the project, the distance between points i_2,2_ and i_2,10_ was set (560 mm) (Figure 2).

In all modeling, the third radial distortion parameter of the polynomial series (4) was set to zero because the uncertainty had same magnitude as the value. Furthermore, the level of correlation with the second term was generally over 95%.

### 3.2. Experimental Program

For the study of geometric stability and decentering, 4,464 pictures were taken using 372 calibrations (62 per camera). Of these 62 calibrations, the first 20 were conducted at one time without turning off the camera and without operating any control or adjustment. Between each of the following 20 calibrations, the camera was power cycled. This action represents the gathering of zoom lenses within the camera. Finally, we carried out 22 calibrations. The first 20 were intercalated with full extension and retraction of the optical zoom (without turning off the camera), while the last two calibrations were performed after a power cycle.

Using the same images but the special photogrammetric networks explained in section 3.1, 360 new calibrations (60 per camera) were performed to study the change of interior geometry after rotating the camera.

For image processing (subpixel detection of centers), exterior orientation processing and bundle adjustment was performed with Photomodeler 6.0, so the calibration results do not only reflect the geometric accuracy of the camera, but also the accuracy accomplished with Photomodeler software. The process including scaling, orientation and export of model data and was performed by an automated external control script programmed in VBA.

## 4. Results and Discussion

### 4.1. Variation of the Internal Geometry: Principal Distance

**Figure 3.**
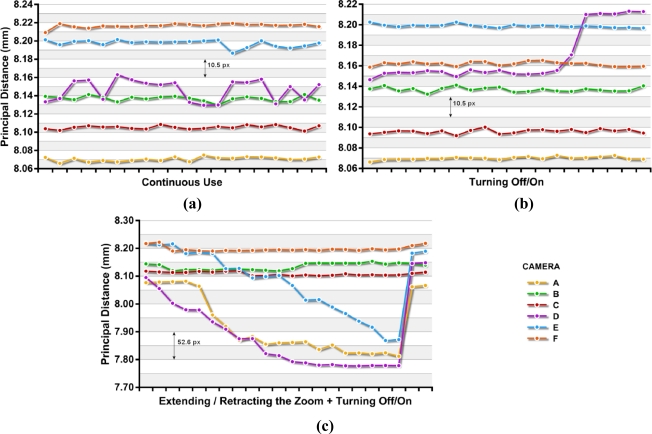
Variations in the principal distance observed in the 372 calibrations performed without shutting down the cameras (a), while applying power cycles (b) and after the extension/retraction of the zoom lens (c).

Figure 3 shows the variations found in the principal distance for the three situations described in section 3.2, continuous use (a), power cycling (b) and extending/retracting the zoom lens (c). The last two calibrations for the extension/retraction of the zoom lens (c) correspond to the principal distances obtained after power cycles. Table 2 and Table 3 summarizes the standard deviations and the maximum variations obtained from each camera in the 20 modeling runs, not including the last two runs in Figure 3 (c).

In all cases for continuous use and power cycling, all cameras except unit D appeared to be stable with standard deviations in their principal distance smaller than 5 μm (2.6 px). These deviations represent less than 0.07% of nominal focal length (7.90 mm). It is noteworthy that the deviations obtained in the off/on tests in four of the cameras are less than those obtained for continuous use tests, although the differences were very small.

Camera D shows unstable behavior, resulting in deviations four to five times higher than the other cameras during continuous use testing. Further major systematic changes were observed in the tests with power cycles. This arbitrary behavior could indicate a mechanical problem in the lens system that does not affect the basic functionality of the camera but certainly limits the usability of the unit for photogrammetric purposes.

After the extension and retraction of the zoom, there is an important and progressive reduction of the principal distance in three cameras. This shortening is recovered after power cycling, as shown in Figure 3c.

The smallest variations were obtained for camera A (6.6 μm, 3.5 px) and C (8.4 μm, 4.4 px) for continuous use and also while applying power cycles. These results are slightly less favorable but comparable to those obtained by Läbe (3 μm) in a Kodak DCS 460 [20] with a 24 mm lens and to those also obtained by Peipe [21] with a D7 Rollei Metric (3 μm) with a 7 mm lens. With camera D much higher variations were reached; 33.6 μm (17.7 px) for continuous use, 66.7 μm (35.1 px) applying power cycles and 317.8 μm (167.3 px) with extension and retraction of the zoom lens. In camera E, with extension and retraction cycles of the zoom lens, differences are achieved up to 351.1 μm, (184.8 px) which represents a variation of 4% of the nominal focal length.

**Figure 4.**
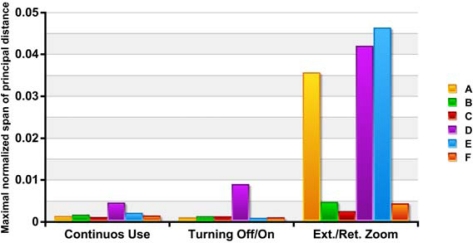
Maximum normalized range of changes in principal distance c. The spans are normalized with the image width.

Figure 4 shows the changes in principal distance refer to the image width: (*c*_max_ − *c*_min_) / *w*. The values obtained in continuous use and with applying off/on cycles, with the exception of camera D, are the same order of magnitude as those cited for Läbe for a Sony DSC V1 with 1x zoom factor [20].

### 4.2. Variation of the Internal Geometry: Position of Principal Point

**Figure 5.**
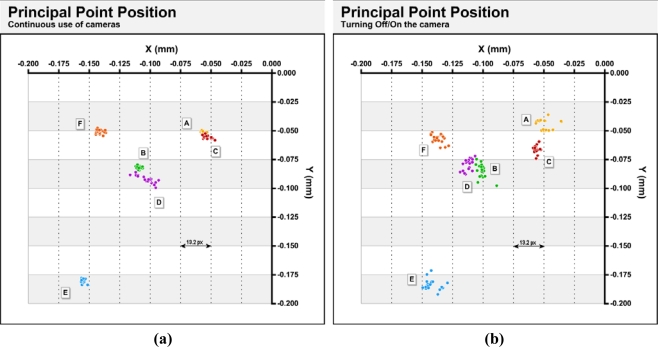

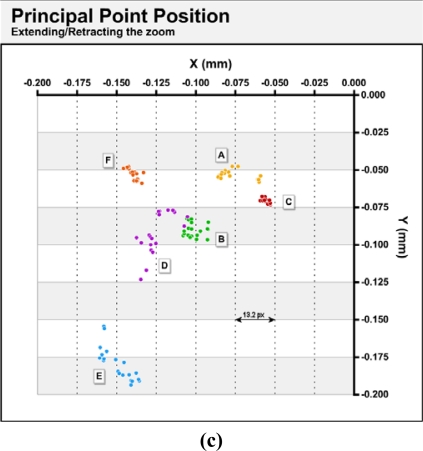
Principal point positions in the 372 calibrations done continuously (a), using power cycling (b) and after the full extension/retraction of the zoom (c).

Figure 5 shows the variations found in the principal point’s positions for the three situations described in section 3.2; continuous use (a), power cycling (b) and extending/retracting the zoom lens (c). In the figure, the origin coincides with the geometric center of the sensor.

As shown in Figure 5, the most stable position of the principal position is obtained, as expected, with continuous use of the cameras. For this case, the X and Y ranges are, except for camera D between 5–10 μm (3–5 px) (Table 4).

After power cycles, dispersions between 6 and 23 μm (3 and 14 px) are obtained. After the cycles of extension and retraction of the zoom lens, greater ranges are obtained and have values between 6 and 39 μm (3 and 21 px). Again, there are differences between the various cameras. Camera C has the lowest dispersion in the 3 situations and a maximum range of 15 μm (6 px) difference. The D unit has a large dispersion, with ranges between 12 and 46 μm (6 and 24 px) (Table 4). All cameras have the principal points in the third quadrant of the sensor. Distances from the geometric center of the sensor vary from a minimum of 70–85 μm (37–45 px) in cameras A and C to a maximum of 235 μm (124 px) in camera E. Considering that the diagonal of a sensor quadrant is 4.75 mm (2500 px), these offsets are between 1.5 and 5%.

### 4.3. Variation of the Internal Geometry: First Radial Distortion Parameter

**Figure 6.**
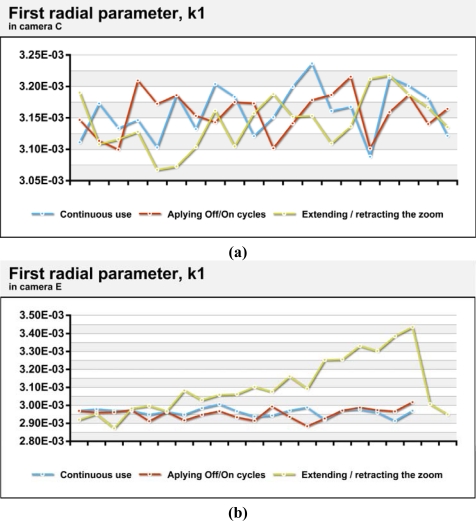
First radial distortion parameter obtained in camera C (a) and E (b) for the 372 calibrations done continuously, by power cycling and after the extension/retraction of the zoom lens.

Figure 6 (a) shows the variations in the first coefficient of radial distortion in camera C, a camera with more stable geometry. In contrast, Figure 6 (b) shows the variations in camera E, as an example of the most geometrically unstable camera among those tested. As seen in the values from camera C, there are no strong trends in the data and the maximum range is less than 5% even for calibrations performed after cycles of extension and retraction of the zoom. In the camera E tests, differences between the maximum and minimum values are up to 20% for calibrations performed after cycles of extension and retraction of the zoom. This is due to the systematic nature of the difference, analogous to the differences in principal distance. The standard variations in the value of the first parameter of radial distortion in situations of continuous use and power cycling are approximately 3 × 10^−5^ (Table 5), which represents 1% relative variation on the mean, a high value but logical given the high correlation between the different parameters of distortion. Camera D showed variations which were significantly higher. Cameras with systematic variations in geometry during extension/retraction of the zoom (D, E and F) reached differences that were over 15% of the average value.

### 4.4. Variation of the Internal Geometry with Rotating the Cameras

Previous investigations have shown that principal point movement in digital cameras is a real phenomenon. Rotating the camera can produce physical movement of the CCD sensor [10] or other movements associated with the effect of the gravity on the lens [13]. If the variations are important may be necessary or convenient to take into account, during a field calibration, the position will have a camera in their subsequent use.

**Figure 7.**
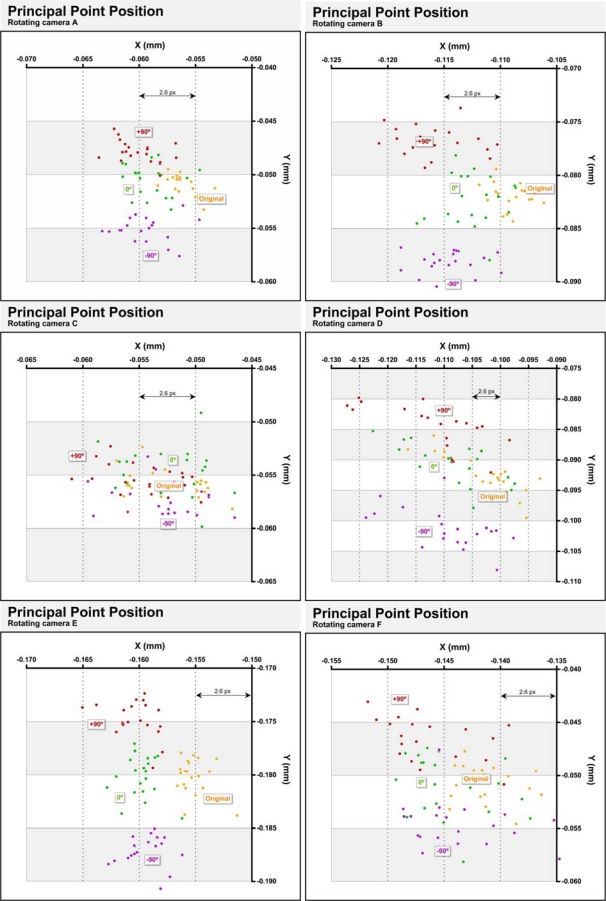
Principal point positions considering the rotating of the cameras.

Figure 7 compares the positions of the principal point in four cases and the six cameras tested. The 20 yellow dots correspond to the positions calculated using the full photogrammetric network explained in paragraph 3.1, with 12 photographs. These points are, therefore, the same as in Figure 5. The green dots are the positions of the principal point obtained from the subset of photographs consisting of the four photographs taken with the camera sub horizontal plus two photographs taken with the camera rotated 90° and −90°. Items in red are the positions obtained from the subset of photographs consisting of four photographs taken with the camera rotated 90° clockwise plus two opposite photographs taken with the camera sub-horizontal. The purple dots indicate the positions of the principal point calculated from the subset of photographs consisting of four photographs taken with the camera rotated −90° plus two photographs taken with the camera sub-horizontal.

Because the results do not only reflect the geometric accuracy of the camera, but also the accuracy accomplished with Photomodeler software, is not possible attribute all variations to physical changes in the cameras. However, considerable differences between the cameras would show that at least in part, changes are independent of the software. Thus, camera C has the smallest differences being all positions in the range of 15 μm (8 px) along the axis X and 10 μm (5 px) μm along the axis Y. Except chamber D, which presents an abnormal behavior, the rest of the cameras show a similar behavior: along the X-axis displacements between 5 and 10 μm (3 and 6 px) are observed with respect to the complete photogrammetric network. Along the Y axis more pronounced displacements are observed which are dependent on the direction of rotation of the camera, which shows that changes in the camera’s internal geometry are induced by the gravitational action. Table 6 shows the principal distances obtained in the various subnets. In parentheses are shown the differences with respect to those obtained by the complete network. Table 7 shows the standard deviations of several parameters obtained in the four subnets. This table shows the results of a single camera A calibration for each network.

As shown in Table 6 there are important differences regarding the use of full photogrammetric networks. The differences between different networks are greater than those found between successive calibrations (Figure 3a and Table 3).

Marking residuals are greater with complete network (Table 7) as a result of sensor – lens change. However almost every parameter are more precisely calculated with the complete network. The reason for this apparent contradiction is that the complete network is symmetrically balanced.

According to the objectives proposed in this paper can conclude the convenience of calibrating this type of camera with photos taken primarily in the same position in which the camera will be used.

### 4.5. Distortions Produced in the Tested Cameras

**Figure 8.**
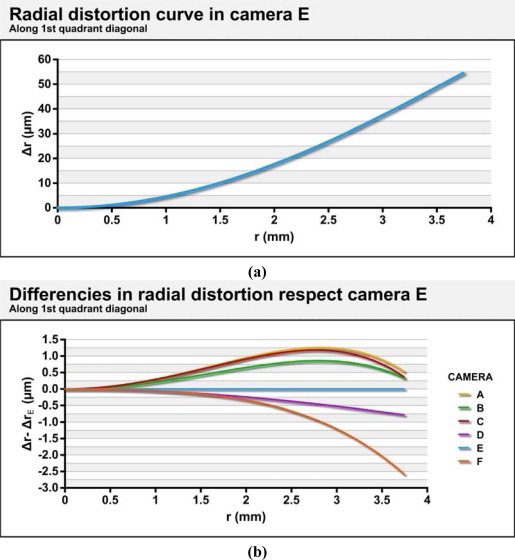
Radial distortion curve for the camera E (a). Curves of radial distortion in the rest of the cameras with respect to camera E (b).

**Figure 9.**
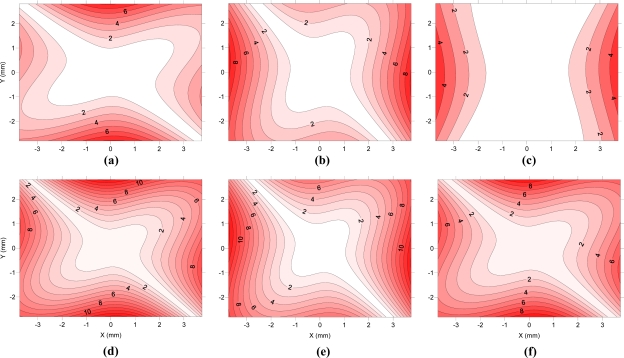
Radial component of the decentering distortion (in microns) in cameras A (a), B (b), C (c), D (d), E (e) and F (f).

**Figure 10.**
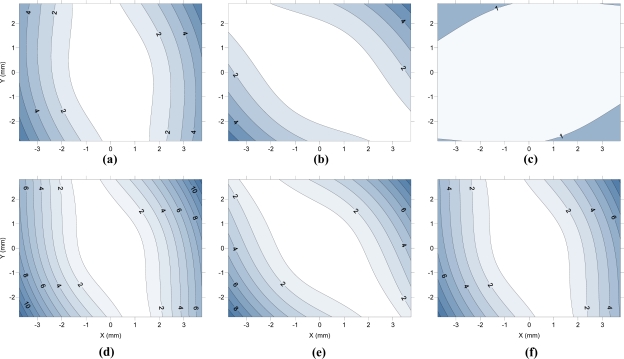
Tangential component of the decentering distortion (in microns) in cameras A (a), B (b), C (c), D (d), E (e) and F (f).

Figure 8 (a) shows the value for radial distortion (including k_1_ and k_2_) of camera E along the diagonal of the first quadrant. The data used are the averages of the parameters obtained in calibrations performed continuously. Figure 8 (b) shows the differences in radial distortions for the remaining cameras based on those obtained from the camera E. Figure 9 displays the radial component of the decentering distortion and is plotted along the entire sensor, while Figure 10 displays the tangential component for the six cameras studied. The values used for preparation of the figures are averages of the parameters obtained in modeling with continued use of the cameras.

As shown in the figures, all of the cameras show a similar radial distortion profile. Camera F has the largest difference from the others (−2.58 μm). The differences found between different cameras represent less than 5% of the radial distortion of either.

As for decentering distortion, five of the six cameras show a very similar orientation for the axis of least distortion, which may indicate a systematic cause (failure to design or manufacture) of decentering. Only camera C has a different orientation for lower decentering distortion, and it is also the camera with less decentering distortion.

We note that the radial component of the decentering distortion reaches values above 12 μm in camera D, while camera C barely reaches 5 μm. A similar trend occurs in the tangential component, with camera D demonstrating values above 12 μm while the camera C did not reach 2 μm in any area of the sensor. The decentering distortion values are lower than those obtained by radial distortion, but the differences are not sufficient to justify their disregard.

### 4.6. Photogrammetric Precision Regarding the Instability and Decentering of Cameras

Table 8 and Table 9 show the marking residuals obtained from the six cameras in the three situations described in section 3.2. Each cell represents the average of 20 calibrations. The data in Table 8 are calculated from the average of the marking residuals through all points, while the data in Table 9 are made using the maximum marking residual obtained at any point.

Lower marking residuals in a calibration generally represent better modeling of a camera achieved by better photogrammetric network design. When the calibrations are done under uniform conditions (using the same photogrammetric network) and the results are constant and reproducible, greater photogrammetric precision (marking residuals up to 80% lower) can be related to greater sharpness of the images obtained. This is indicative of a higher quality camera or of limitations in modeling the decentering distortion.

The lowest marking residuals are systematically obtained in cameras A and C. In these cameras the decentering distortion is lower (Figure 9 and Figure 10) and their principal points are the closest to the geometric center of the sensor (Figure 4). On the other hand the worst precision is obtained with camera D which is the greater decentering distortion introduced. In view of these results it can be deduced that there is a direct relationship between the level of decentration of the lenses and lower accuracy in a camera, not being effective enough compensation used to model this distortion (6). In addition, cameras that have fewer decentering distortion also have less offset of the principal point which can be used as an initial criterion to assess the level of decentration between similar cameras. But using this criterion should be conservative as the principal point offset may also be due to a lateral displacement of the sensor.

## 5. Summary and Conclusions

One of the six cameras analyzed shows significant geometric instability, even without moving the objective lens. This instability may be due to faulty construction in the lens system that does not prevent the operation of the camera but does limit its potential for metrics use. The rest of the cameras have shown that variations of the internal geometry, during continuous use or after applying cycles off/on are comparable to those obtained with other cameras used in photogrammetric applications of medium or low accuracy.

Variations in the geometry of the camera after the extension and retraction of the zoom lens are important in the six cameras analyzed. The principal distance undergoes major systematic changes in three of the cameras, while the dispersion in the position of principal point shows an increase that is significant relative to the results obtained during continuous use of the cameras.

It has been shown that decentering distortion, while less than the radial distortion, cannot be neglected. The decentering in all six lens-sensor systems are not arbitrary. The six principal points are located in the same quadrant, and in five of the cameras, the direction of the maximum tangential component is in approximately the same direction, indicating a systematic cause of decentration.

Less photogrammetric precision (estimated from marked residues) was observed in the cameras that have the highest level of lens decentration. The reasons for this decrease in precision may be due to the worse quality of the pictures (less sharpness) and/or due to the limited effectiveness of used decentering distortion correction.

These results suggest that during photogrammetric use of these kind of cameras (digital compact cameras) it is advisable (or necessary) to perform a new modeling for a camera after the voluntary or involuntary action of the zoom lens. However, for the photogrammetric accuracy expected from this kind of camera, it may be permissible to not model the camera between uses, including uses after power cycles. During the modeling of the cameras it may be advisable to consider the future position of the camera as they have been proven non-negligible systematic variations of the internal geometry of cameras when they are rotated. Finally, given the differences found for the stability and decentration between cameras that are theoretically equal, a recommended criterion for choosing a camera among several equals can be the minor offset in the principal point.

## Figures and Tables

**Photograph 1.**
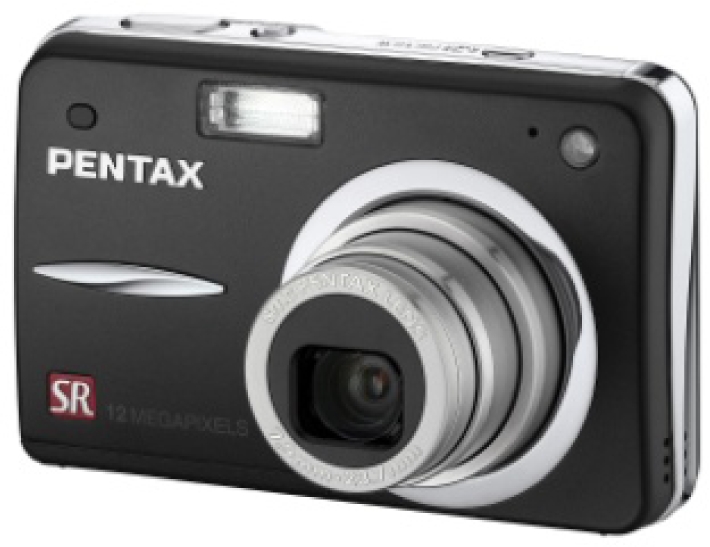
Pentax Optio A40 camera. (From www.pentax.co.jp).

**Photograph 2.**
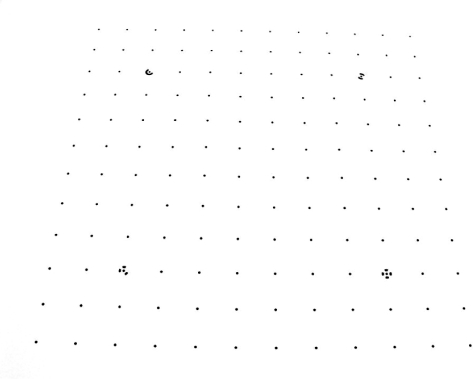
Plane point field used during calibration of the cameras.

**Table 1.** Technical characteristics of cameras used in the measurement system (from www.dpreview.com and www.pentax.co.jp).

**Feature**	**Pentax Optio A40**
Effective pixels	4,000 × 3,000
Image ratio w:h	4:3
Sensor pitch	1/1.7 inch, 7.60 × 5.70 mm, 0.43 cm^2^
Pixel density	28 MP/cm^2^
Pixel size	1.9 μm × 1.9 μm
Sensor type	CCD
Lens	7 elements in 5 groups (2 dual-sided aspherical elements, 1 single-sided aspherical element)
Focal Length	7.90 mm – 23.7 mm
Sensitivity	ISO 50–1600
Aperture	F2.8-F5.4
Shutter speed	4 s-1/2,000 s
File Formats	JPEG (EXIF 2.2)

**Table 2.** Variations of the principal distance, expressed by standard deviations obtained from the six cameras tested in the 20 modeling runs.

**Principal distance’s standard deviations**	**CAMERA**											
	**A**		**B**		**C**		**D**		**E**		**F**	
	**μm**	**px**	**μm**	**px**	**μm**	**px**	**μm**	**px**	**μm**	**px**	**μm**	**px**
Continuous use	2.4	1.3	2.9	1.5	2.0	1.1	11.6	6.1	3.8	2.0	2.2	1.2
Turning Off/On	1.5	0.8	2.3	1.2	2.0	1.1	25.8	13.6	1.6	0.8	2.1	1.1
Extending/Retracting the Zoom	103.1	54.3	12.3	6.5	6.5	3.4	104.7	55.1	116.1	61.1	8.2	4.3

**Table 3.** Maximum variations of the principal distance, obtained from the six cameras tested in the 20 modeling runs.

**Principal distance’s maximum variations**	**CAMERA**											
	**A**		**B**		**C**		**D**		**E**		**F**	
	**μm**	**px**	**μm**	**px**	**μm**	**px**	**μm**	**px**	**μm**	**px**	**μm**	**px**
Continuous use	9.0	4.8	11.6	6.1	7.2	3.8	33.6	17.7	14.9	7.8	10.0	5.2
Turning Off/On	6.6	3.5	8.8	4.6	8.4	4.4	66.7	35.1	5.8	3.0	6.7	3.5
Extending/Retracting the Zoom	269.8	142.0	34.8	18.3	18.1	9.5	317.8	167.3	351.1	184.8	32.2	16.9

**Table 4.** Maximum variations in the position of the principal point.

**Camera**	**X-Range**						**Y-Range**					
	**Continuous**		**Off / On**		**Ext. / Retr.**		**Continuous**		**Off / On**		**Ext. / Retr.**	
	**μm**	**px**	**μm**	**px**	**μm**	**px**	**μm**	**px**	**μm**	**px**	**μm**	**px**
**A**	5	3	21	11	27	14	4	2	14	7	10	5
**B**	6	3	17	9	16	8	5	3	23	12	14	7
**C**	10	5	6	3	6	3	6	4	15	8	6	3
**D**	23	12	12	6	32	17	13	7	16	8	46	24
**E**	5	3	20	11	25	13	6	3	21	11	39	21
**F**	8	4	15	8	13	7	7	4	13	7	11	6

**Table 5.** Standard deviations of the first parameter of radial distortion obtained from the six cameras tested over the three situations studied. The value in parentheses is the relative standard deviation to the average value.

	**CAMERA**					
	**A**	**B**	**C**	**D**	**E**	**F**
Continuous use	1.62E-05 (0.51%)	3.54E-05 (1.14%)	3.96E-05 (1.25%)	7.01E-05 (2.4%)	2.20E-05 (0.74%)	2.89E-05 (0.98)
Power cycling	3.24E-05 (1.03%)	2.99E-05 (0.97%)	3.34E-05 (1.06%)	3.62E-05 (1.24%)	3.15E-05 (1.07%)	2.98E-05 (1.03%)
Extending/Retracting the Zoom	1.61E-04 (4.74%)	2.40E-05 (0.77%)	4.18E-05 (1.33%)	2.07E-04 (6.21%)	1.59E-04 (5.1%)	2.99E-05 (1.03%)

**Table 6.** Principal distances according to the different subnets.

**Principal distances (μm)**	**CAMERA**					
	**A**	**B**	**C**	**D**	**E**	**F**
**Complete Network**	8.071	8.137	8.105	8.146	8.197	8.217
**0°**	8.068 (−3)	8.133 (−4)	8.090 (−15)	8.167 (+21)	8.196 (−1)	8.200 (−17)
**90°**	8.073 (+2)	8.128 (−9)	8.091 (−14)	8.152 (+6)	8.187 (−10)	8.204 (−13)
**−90°**	8.061 (−10)	8.145 (+8)	8.097 (−8)	8.180 (+34)	8.208 (+11)	8.206 (−11)

**Table 7.** Standard deviations obtained with the different subnets.

**Standard deviations**	**Complete Network**	**0°**	**−90°**	**+90°**
**Principal distance (mm)**	3.0E-04	5.0E-04	6.1E-04	5.4E-04
**Pp_x_ (mm)**	1.9e-04	2.6E-04	5.0E-04	4.5E-04
**Pp_y_ (mm)**	2.1e-04	5.0E-04	4.4E-04	3.8E-04
**k_1_**	5.9E-06	9.4E-06	1.1E-05	1.3E-05
**k_2_**	3.3E-07	5.6E-07	7.6E-07	1.2E-06
**P_1_**	7.3E-7	9.8E-07	1.8E-06	1.6E-06
**P_2_**	7.6E-07	1.8E-06	1.6E-06	1.4E-06
**Global RMS (px)**	0.073	0.057	0.055	0.039
**Maximun RMS (px)**	0.165	0.180	0.129	0.065
**Minimum RMS (px)**	0.040	0.022	0.017	0.015

**Table 8.** Global marking residuals (in pixels) obtained in the 372 calibrations performed in all six cameras analyzed.

**Global RMS (px)**	**CAMERA**						**Average**
	**A**	**B**	**C**	**D**	**E**	**F**	
Continuous use	0.078	0.130	0.097	0.143	0.127	0.111	**0.114**
Turning Off/On	0.084	0.120	0.097	0.134	0.131	0.105	**0.112**
Extending/Retracting the Zoom	0.090	0.131	0.098	0.152	0.136	0.103	**0.118**
**Average**	**0.084**	**0.127**	**0.097**	**0.143**	**0.132**	**0.106**	

**Table 9.** Maximum marking residual (in pixels) obtained in the 372 calibrations performed in all six cameras analyzed.

**Maximum Residual (px)**	**CAMERA**						**Average**
	**A**	**B**	**C**	**D**	**E**	**F**	
Continuous use	0.247	0.360	0.288	0.446	0.383	0.371	**0.349**
Turning Off/On	0.265	0.307	0.285	0.364	0.432	0.320	**0.329**
Extending/Retracting the Zoom	0.316	0.361	0.302	0.488	0.424	0.302	**0.366**
**Average**	**0.276**	**0.342**	**0.292**	**0.433**	**0.413**	**0.331**	
